# Highly Corrugated Ni Films Electrodeposited onto Boron Doped Diamond Electrodes for Alkaline Water Electrolysis

**DOI:** 10.1021/acselectrochem.5c00319

**Published:** 2025-10-20

**Authors:** Alexander W. Black, Paul W. May, David J. Fermin

**Affiliations:** School of Chemistry, 1980 University of Bristol, Bristol BS8 1TS, UK

**Keywords:** boron-doped diamond, NiCu alloys, electrodeposition, HER mechanism, alkaline electrolysis

## Abstract

Ni-based electrocatalysts are among the most active materials for the hydrogen evolution reaction (HER) in alkaline media. In this work, we demonstrate the ability to use films of boron-doped diamond (BDD), a stable and corrosion-resistant electrode material, as a support for highly textured Ni films. Our approach is based on the electrodeposition of NiCu alloy thin-films, followed by electrochemical dealloying. The structure and composition of the electrocatalysts were characterized using scanning electron microscopy, X-ray diffraction, and X-ray photoelectron spectroscopy. In pH 13 KOH, a dealloyed Ni catalyst corresponding to an initial NiCu composition of 68% Ni, gave a HER overpotential at 10 mA cm^–2^ of 152 mV. With further analysis, we show that the rate of HER is 2^nd^ order with respect to the number of Ni active sites, and that the kinetics are limited by the surface diffusion of adsorbed intermediates. Two Tafel slopes are additionally observed, suggesting a change in HER mechanism and intrinsic activity at dealloyed Ni catalysts.

## Graphical abstract

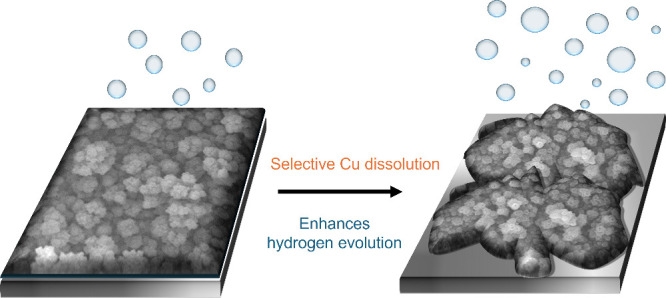

## Introduction

Alkaline water splitting is of interest as a method to generate green hydrogen, however the cathodic process, the hydrogen evolution reaction (HER), remains a limiting factor to performance. Various models have been proposed to explain the pH dependence of the HER, and how best to enhance it in basic electrolytes including: the ‘bifunctional’ approach;^1,2^ the potential of zero free charge;^3,4^ and finally the H-binding energy concept.^5^ A multitude of materials have been reported as active for HER and this topic has been reviewed several times.^6–11^ Generally, the most active catalysts are based on Ni, or on Ni compounds incorporating other first-row transition metals and/or p-block elements.^6,12^ In addition to its activity, Ni is low-cost and Earth abundant. However, the sustainable mining and processing of Ni ore could be compromised due to rapidly increasing demand.^13^ Consequently, utilization of Ni in areas such as interfacial catalysis requires low loadings and high specific surface areas.

A less commonly discussed aspect of electrode design is the role of the support material. sp^2^ type carbons are the most common choice but their tendency to corrode is well documented,^14–16^ including at conditions such as those encountered in both catalyst layers of alkaline water electrolysis.^14,17–20^ An alternative catalyst support that could be considered is with boron-doped diamond (BDD) as the carbonaceous material. Diamond can be doped to near metallic levels of conductivity, is mechanically robust and highly resistant to corrosion.^21–23^ Diamond-based materials have been investigated as electrocatalyst supports but mainly in acid.^16,24–28^ Reports also exist in alkaline electrolytes, but only for powders.^29,30^ BDD was observed to corrode at much slower rates than sp^2^ carbons, therefore indicating that there are significant advantages to be gained by using BDD as the electrocatalyst support. However, none of these studies achieved continuous metallic layers onto the BDD support with adhesion strong enough for electrocatalytic applications.

In the present work, we demonstrate that co-deposition of Cu and Ni followed by electrochemical dealloying is an effective route to generate adherent highly corrugated Ni films onto BDD electrodes, with high electrocatalytic activity towards HER in alkaline electrolytes. Building upon the work by Sun et al.,^31^ and Chang et al.,^32^ we achieved for the first time continuous and stable Ni elecrodeposits at BDD with tunable corrugation upon electrochemical dealloying. The study initially investigates the electrochemical responses of O-terminated BDD electrodes. BDD electrodes were subjected to a mild stability test, and the occurrence of any corrosion was studied with Scanning Electron Microscopy (SEM) and Raman spectroscopy. The rough and porous Ni films were characterized with SEM, X-ray Diffraction (XRD) and X-ray Photoelectron Spectroscopy (XPS) both before and after dealloying. NiCu deposition conditions of NiCu were varied to maximise the Electrochemical Surface Area (ECSA), HER activity, and to understand the mechanism of HER on dealloyed (DA) Ni electrodes. Our analysis also uncovers a second-order dependence of the HER kinetics on the density of active Ni surface sites estimated from the pseudocapacitive responses arising from the formation of α-Ni­(OH)_2_.

## Experimental Section

### Chemicals

Nickel­(II) sulfate hexahydrate, (>99%), copper­(II) sulfate pentahydrate (>99%), nickel­(II) chloride hexahydrate (>99%), boric acid (>99.5%) and KOH (>85%), were all purchased from Sigma-Aldrich, UK and used without further alteration. Solutions were prepared using ultrapure Milli-Q water (Merck, Germany), 18.2 MΩ cm and TOC < 5 ppb.

### Electrodes

Boron-doped diamond electrodes were grown onto degenerate (100) p^++^ Si wafers (University Wafer, USA) using Hot Filament Chemical Vapor Deposition (HF-CVD) in a process that has been described previously.^33^ Briefly, the Si substrates were manually abraded with 1-3 μm diamond powder (Van Moppes Ltd.), the powder was then wiped off the surface using a cotton-bud soaked in isopropanol and dried in a stream of dry N_2_. The sample was then placed into the steel CVD chamber containing 20 Torr of a gas mixture of 1% H_2_/CH_4_ plus 1000 ppm B_2_H_6_. Tantalum filaments situated 5 mm above the Si substrate surface were electrically heated to ∼2400 K, which fragmented the gas mixture into reactive radicals and ions, and heated the substrate to ∼900 °C, causing the diamond film to deposit onto the substrate. This CVD process continued for 6 h, producing a microcrystalline diamond film ∼ 3 μm thick with near metallic conductivity, as demonstrated elswhere.^34^

After diamond growth, the BDD electrodes used for stability testing were exposed to ozone generated by a Jelight UVO cleaner 42-220 (Jelight Company, USA) for 30 min to produce O-terminated surfaces, giving pristine electrodes with no history. As grown BDD is H terminated, and UV-ozone treatment leads to a surface composed primarily of ketone and hydroxyl groups.^35^ Alternatively, BDD electrodes could be reused by soaking in aqua regia for at least 3 h. Aqua regia treatment gives a surface functional group composition similar to that produced by UV–ozone treatment.^35,36^ Electrical contact was made from the rear of the electrode, through the Si substrate, by abrading the Si with SiC paper and application of gallium–indium eutectic (eGaIn, Sigma-Aldrich, UK). The high conductivity of the p^++^ Si, nominal sheet resistance between 0.001 – 0.005 Ω cm, enables a good Ohmic back contact. An 8 mm-diameter disc of this electrode was exposed to the electrolyte, and the remainder of the area insulated using a custom PEEK sample holder.

For electrodeposition experiments, a glassy carbon rod was used as the counter electrode (CE) and Ag/AgCl in a 3 M KCl storage solution, separated from the electrolyte by a porous glass frit, as the reference electrode (RE). For experiments in KOH, a graphite rod and Hg/HgO (mercury/mercury oxide, MMO) in a 0.1 M KOH storage solution were used as CE and RE, respectively. The MMO used in this work was calibrated against another MMO used only for this purpose, Potentials were converted to the reversible hydrogen electrode scale (RHE) using 0.141 V as *E* ^0^ for Hg/HgO in 0.1 M KOH.^37^

### Equipment

All electrochemistry experiments were performed with a PGSTAT302N (Autolab, UK) with added FRA32M module and controlled with NOVA 1.11 software (Autolab, UK). SEM micrographs were obtained on a JEOL JSM-IT300, operated at an accelerating voltage of 15 kV with a working distance of 10 mm. Energy Dispersive X-ray Spectroscopy (EDS) data were collected using an X-Max 80 mm^2^ EDX detector and analysed with AZtec software, both from Oxford Instruments, UK. XRD was performed using a Bruker D8 Advance powder X-ray Diffractometer using Cu Kα radiation in the range 20-80° with a step size of 0.02° and a step time of 2 s, whilst rotating the sample at 30 rpm. Raman spectra were collected with a Renishaw 2000 laser Raman spectrometer fitted with 3 lasers with wavelengths at 325, 514, and 785 nm, with the 514 nm laser used in these experiments. XPS information was obtained with a NanoESCA II and an Argus XPS analyser, both from Scienta Omicron, Sweden. Spectroscopy was performed with a monochromatic Al Kα source (1486.7 eV) operating at 15 kV and 18 mA (270 W), with pass energies of 100 and 50 eV for survey and high-resolution spectra, respectively. Survey spectra were acquired at the start and end of each sample illumination to check for X-ray beam induced modifications. Data analysis was performed using CasaXPS software v.2.3.25PR1.0,^38^ a Shirley background was added to the spectra and the relevant peaks were integrated to give the total intensity associated with each element. For Ni and Cu, this was 2p_3/2_. Compositional information was obtained by normalising the area by the appropriate relative sensitivity factor (RSF), measured for the specific instrument, multiplied by an inelastic mean free path correction, λ, where λ = (KE–BE)^0.79^ and KE is the beam kinetic energy, BE is the binding energy of the relevant element and 0.79 is a constant obtained from the literature.^39^

### Methodology

All glassware was cleaned by firstly immersing overnight in a solution of 0.5 M H_2_SO_4_ and 1 g L^–1^ KMnO_4_, followed by rinsing with dilute piranha solution and then soaking in boiling ultrapure water four times. Glassware was stored in ultrapure water between experiments. All solutions were degassed by bubbling argon through them for at least 20 min *prior* to the start of the experiment and an argon blanket was maintained for the duration. Electrochemical impedance spectroscopy was used to estimate the uncompensated resistance of the systems, with HER activity results being corrected for 90% of the measured value.

NiCu electrodeposition was performed in a solution containing 0.2 M NiSO_4_, 0.01 M CuSO_4_ and 0.5 M H_3_BO_3_ with conditions described in the text. Electrochemical dealloying was carried out at 0.6 V vs. Ag/AgCl for 600 s immediately after the deposition was finished. Experiments in basic media were performed at pH 13 after accounting for the activity of KOH in water, which typically corresponded to solutions of 0.128 M KOH,^37^ measured with a HI-5222 pH meter (Hanna Instruments, UK). After dealloying, the samples were cycled in pH 13 KOH in the region of α-Ni­(OH)_2_ formation and reduction until a reproducible voltammogram was observed. The final scan was used to estimate the electrochemical surface area (ECSA) using a monolayer charge of 514 μC cm^–2^ and eq 1,^40^

$$A_{\mathrm{ECSA}}=\frac{\int_{E_{1}}^{E_{2}}idE}{514\;\mu C{\mathrm{cm}}^{-2}\nu }$$ 1

where *A*_ECSA_ is the electrochemical surface area, the integral on the numerator represents the area under the voltammogram of the peak associated with α-Ni­(OH)_2_ formation and ν is the scan rate.

Tafel analysis was performed by potential steps, the desired potential was applied for 30 s and the current at the end of the step used. After each measurement the electrode was refreshed by rinsing with water and applying a potential of 0 V vs. RHE.

## Results and Discussion

### Electrochemistry of Boron-Doped Diamond in Basic Media

Figure 1a shows a typical SEM image of the BDD electrode surface, featuring faceted micrometer-sized BDD grains forming a highly compact layer. For these experiments, pristine diamond electrodes were used with an oxygen functionalized surface achieved by UV-ozone treatment. Further details can be found in the Experimental Section. Linear sweep voltammograms at 5 mV s^–1^ of the BDD in pH 13 KOH are displayed in Figure 1b, showing the extent of the electrochemical window under the experimental conditions, as well as the overpotentials for HER and OER. The 2.6 V electrochemical solvent window, defined as the potentials at which the geometric current density reaches ±0.4 mA cm^–2^, is consistent with BDD diamond surfaces with a low degree of graphitic impurities.^41^ Figure 1c contrasts a so-called ‘mild’ stability test at a constant current of ±10 mA cm^–2^ for 8 h in pH 13 KOH at 25°C. When BDD acted as a cathode during stability testing in Figure 1c, the potential quickly decayed by ∼20-25 mV before reaching a plateau for the remainder of the experiment. In the anodic test, the potential change was more significant upon oxygen evolution, increasing by around 1.2 V. These observations suggest that surface modifications are more significant under anodic conditions. However, SEM images of the electrodes (see Figure S1) revealed no microscopic changes in grain morphology. Indeed, we do not observe any changes in morphology at any scale in comparison to a pristine BDD surface, confirming that the structural integrity of the electrode is preserved under anodic and cathodic conditions.

**Figure 1:**
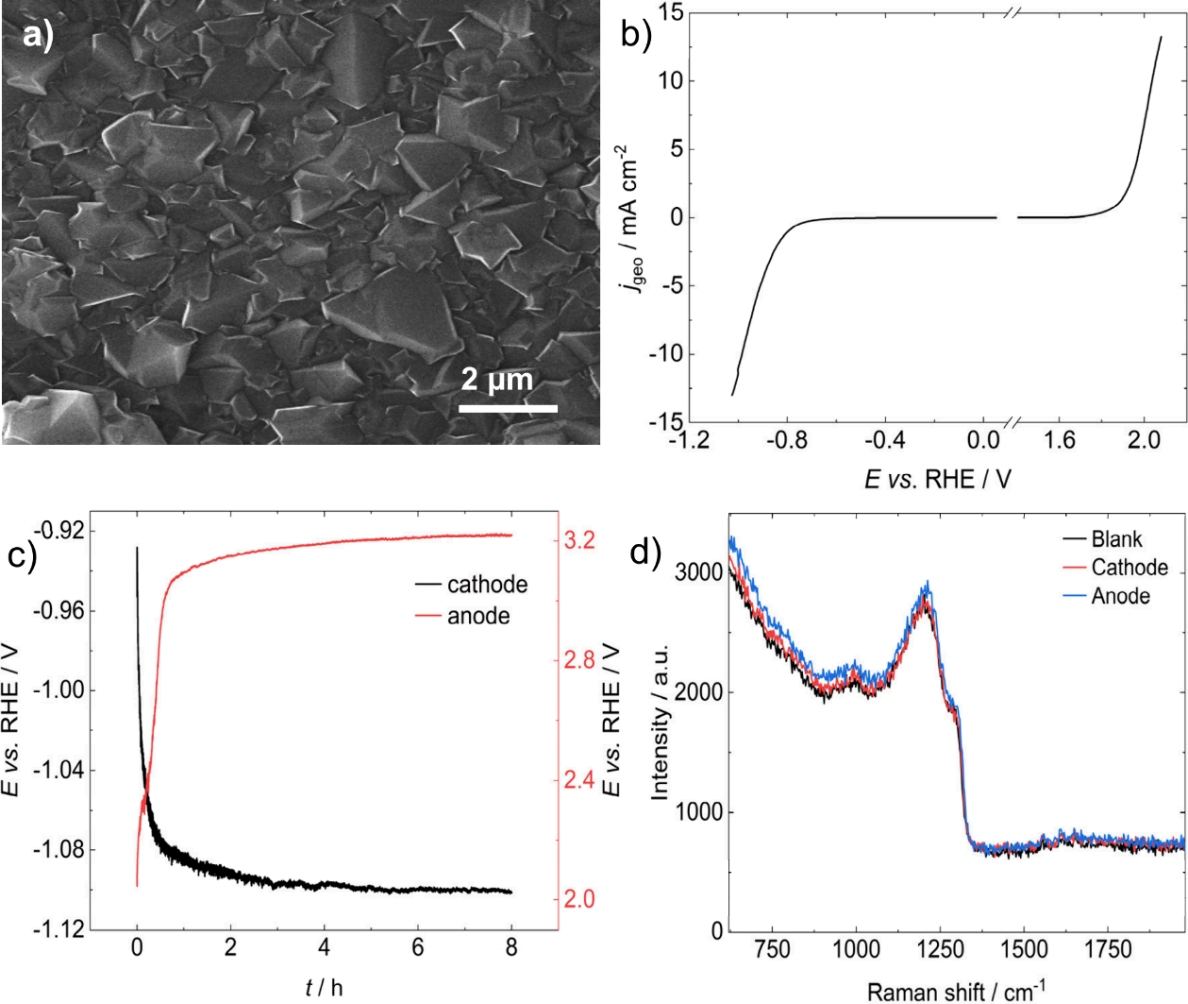
Electrochemical responses of O-terminated polycrystalline BDD electrodes in KOH solution pH 13 at 25°C. a) SEM image of HF-CVD microcrystalline BDD. b) Linear sweep voltammetry at a scan rate of ν = 5 mV s^–1^. c) Chronopotentiograms at anodic and cathodic currents of 10 mA cm^–2^ (geometric area). d) Raman spectra of pristine BDD along with spectra of BDD electrodes after a mild stability test of ± 10 mA cm^–2^ in pH 13 KOH at 25°C for 8 h. Spectra collected with a 532 nm laser.

Figure 1d shows a Raman spectrum of each electrode after the electrolysis, along with that of a pristine BDD surface. The spectrum of the fresh BDD qualitatively appears similar to those reported previously for heavily doped BDD,^33,42,43^ where the broad peak at ∼ 1200 cm^–1^ results from B centres observed when diamond is doped with high concentrations of boron. The Raman mode associated with diamond is now present as a shoulder at ∼ 1290 cm^–1^. The small feature at 990 cm^–1^ is the second order phonon peak from the Si substrate. Crucially, no additional peaks can be observed at wavenumbers higher than 1400 cm^–1^ for all three spectra. The Raman modes of sp^2^ type carbon manifest themselves in the range 1400 to 1800 cm^–1^ and the absence of peaks in this region suggests that no graphitisation and corrosion of the diamond surface takes place during the stability tests. We cannot exclude the possibility that the increase in OER overpotential shown in Figure 1c could be related to the removal of sp^2^ carbon impurities at the surfaces, as reported by other groups.^44^ However, our Raman analysis in Figure 1d does not show any evidence of that to support this hypothesis.

The dimensional stability of BDD electrodes has also been reported at low pH in the seminal reports by Swain and co-workers, confirming that BDD undergoes no changes in morphology. This is in comparison to the substantial microstructural degradation of glassy carbon under water oxidation in acid solutions at room temperature and at 80°C.^45,46^ It should also be mentioned that glassy carbon electrodes also undergoes significant changes in double layer capacitance and microstructure leading to anodic dissolution in alkaline solutions.^47^

### Electrodeposition of High Surface Area Nickel Thin Films

Whilst the electrodeposition of Ni nanoparticles has been reported previously in the literature,^48,49^ there is little evidence of thin film electrodeposition onto BDD. The BDD electrodes used had been treated with aqua regia to give a metal free, O terminated surface. We found it possible to electrodeposit nickel onto BDD from a Watt’s type bath (0.2 M NiCl_2_, 0.8 M NiSO_4_, 0.5 M H_3_BO_3_) and also with an electrochemical dealloying approach based on the co-deposition of Ni and Cu which, as will be shown below, provides a convenient method for the fabrication of rough Ni electrodes with a high surface area. The resulting Ni coatings were stable and adherent under HER conditions. It is important to emphasise that extensive surface oxidation of BDD is key for the adherence of the electrodeposited films.

Figure 2 contrasts the cyclic voltammograms associated with the electrodeposition of Ni and Cu, as well as the NiCu alloy onto BDD diamond electrodes. The electrolyte contained 0.2 M NiSO_4_, 0.01 M CuSO_4_ in 0.5 M H_3_BO_3_. The electrolyte composition is adapted from the work by Sun et al.^31^ The voltammogram in Figure 2a shows the kinetically hindered deposition of Ni, with no cathodic current developing until −0.5 V vs. RHE, where the onset of Ni deposition and HER are observed. Upon reversal of the potential, no evidence of anodic Ni stripping is seen in the potential range investigated. The small anodic peak at 0.48 V vs. RHE is linked to the formation of Ni oxide.^31^ On the other hand, Figure 2b shows the characteristic voltammogram of Cu nucleation and diffusion limited growth with an onset potential of 0.5 V vs. RHE. On the reverse sweep, the deposited Cu strips with an oxidation efficiency (o.e.) of 90%. The co-deposition of Cu and Ni shown in Figure 2c exhibits similar characteristics to the Cu only bath, with a cathodic onset at 0.4 V vs. RHE, and additional current loop at -0.4 V which could be link to Ni electrodeposition. On the reverse scan, there are two anodic peaks associated with the stripping of two different Cu forms.^31,32^ The oxidation efficiency is 72%, with the discrepancy due to HER and Ni electrodeposition.

**Figure 2:**
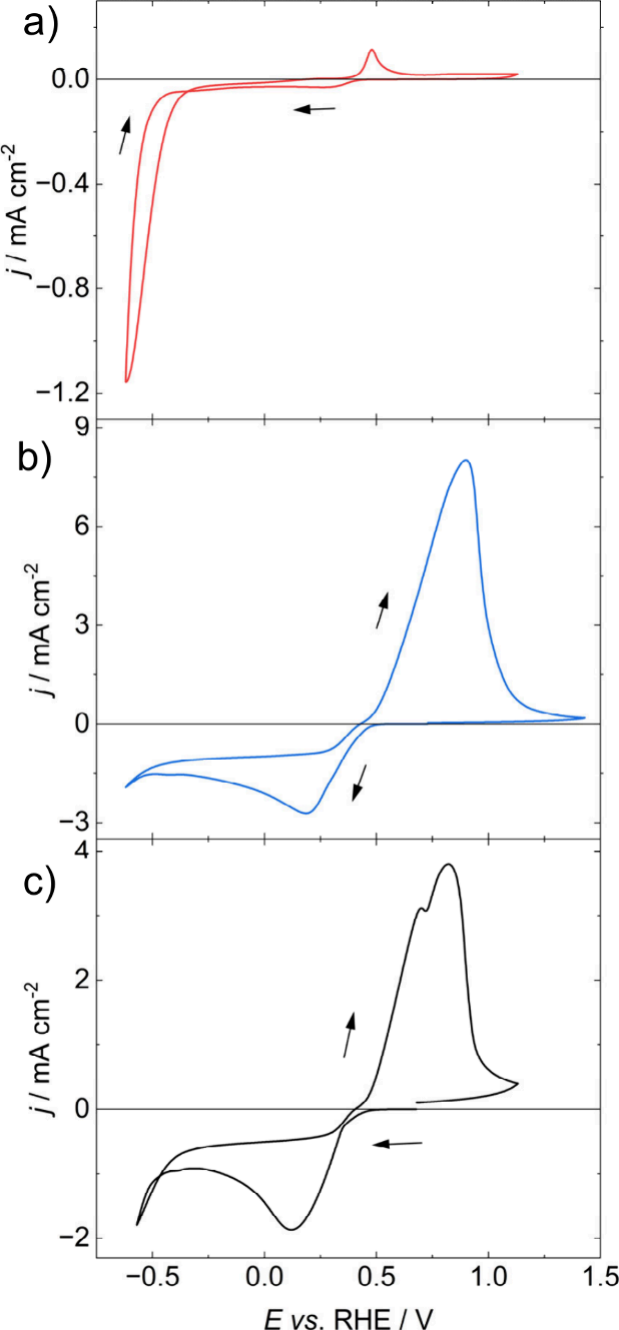
Voltammograms for a BDD WE with radius *r* = 4 mm in an aqueous electrolyte containing voltammograms of a) 0.2 M NiSO_4_, b) 0.01 M CuSO_4_ and c) 0.2 M NiSO_4_, 0.01 M CuSO_4_ and 0.5 M H_3_BO_3_, used as the bath for NiCu electrodeposition and electrochemical dealloying. Scan began at 0.7 V and swept in the direction of the arrows at a rate of *ν* = 50 mV s^–1^, CE: glassy carbon, RE: Ag/AgCl.

This bath was used to electrodeposit the high surface area thin films onto BDD where we found that a deposition potential, *E*_dep_, of −0.42 V vs. RHE and a deposition charge density, *Q*_dep_, of 5.0 C cm^–2^_,_ followed by dealloying at 1.03 V vs. RHE gave deposits with a high degree of texture and roughness. Figure S2 in the Supporting Information shows typical deposition and dealloying transients under these conditions. The deposition current transient shows a slow rising cathodic current over 2000 seconds, which could be rationalised as kinetically controlled growth with an increasing surface area. On the other hand, the dealloying transients show that Cu dissolution is complete after 200-300 s.

Figure 3 shows representative SEM images of the electrodes deposited under the conditions described above of *E*_dep_ = −0.42 V vs. RHE and |*Q*_dep_| = 5.0 C cm^–2^, before and after Cu dealloying. The morphology of the deposit does not change significantly upon dealloying but, similar to previous reports, tubular structures can now be observed.^31^ The deposit has a globular morphology *prior* to dealloying, featuring highly faceted grains with sizes in the range of 100 nm. These highly corrugated structures might be generated by disturbances in mass transport associated with hydrogen evolution during electrodeposition. Cross-sectional SEM images of the electrodes, shown in Figure 3c and 3d, emphasise the rough and porous nature of the deposited catalyst. Figure S3 in the Supporting Information shows that these highly corrugated films form continuously over the diamond surface even after dealloying.

**Figure 3:**
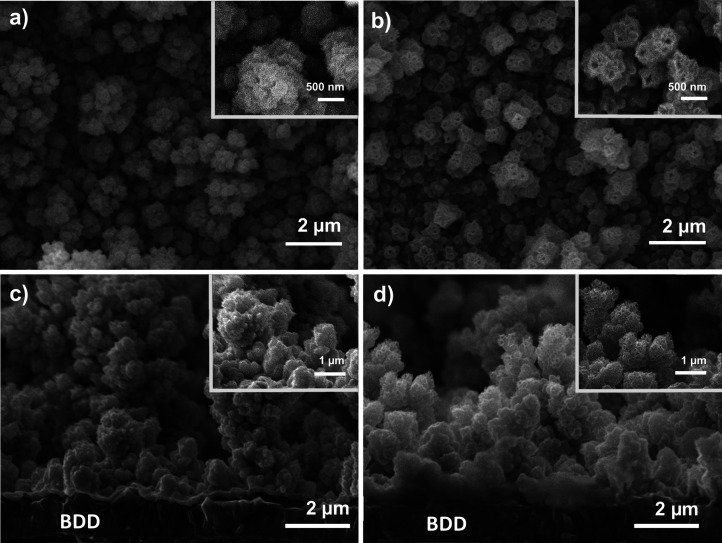
Representative SEM images of electrodeposited NiCu onto a BDD substrate a) before and b) after dealloying, c) cross-section of electrodeposited NiCu, d) cross-section of a dealloyed Ni electrode.

The morphology of the film substantially changes at the microscopic level upon electrochemical dealloying as shown in Figure 3 and Figure S3. The compact and faceted morphology of the alloyed grains (Figure 3a,c) evolves to rather hollowed structures (Figure 3b) exhibiting dendritic-like features (Figure 3d). As shown below, these remarkable features are the centre of the changes in ECSA. Indeed, this is also reflected in SEM images for varying deposition charge densities (Figure S4), which show an increasing degree of disorder in the deposits for larger values of *Q*_dep_.

Figure 4a shows XRD patterns of the NiCu films before and after dealloying. The alloyed electrode displays peaks at approx. 43.9°, 44.6° and 51.9°, along with a broad feature at angles below the peak at 51.9°. The Ni-Cu system forms a solid solution over the entire compositional range at room temperature,^50^ and therefore for a given reflection would be expected to display peaks at positions that are a weighted average of the individual components in accordance with Vegard’s law. However, this appears to not be the case here. This diffractogram suggests that the Ni and Cu are present as both an alloy, and as separate phases. The broad feature between 50–52° may be associated with the diffraction of an NiCu alloy across a range of compositions (see below). Phase segregation of Ni and Cu is then indicated by the presence of peaks at positions corresponding to the individual element, most clearly represented by those at 44.6° and 51.9° for Ni. The formation of separate phases is consistent with previous reports on electrochemical dealloying of NiCu and appears to be a feature of the electrodeposition process.^32^ As discussed further below, our composition analysis shows a gradient of Ni–Cu atomic ratios across the film. After dealloying there is no change in the position of the Ni peaks, the peak at 43.9° decreases significantly in intensity and the shoulder disappears showing the loss of copper.

**Figure 4:**
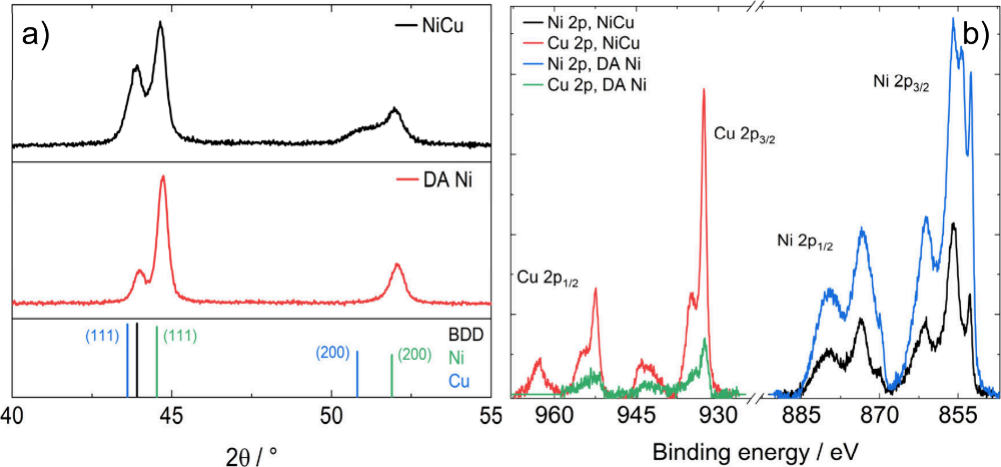
Physical characterisation of NiCu electrodeposited onto BDD before and after dealloying of Cu. a) XRD patterns of NiCu and dealloyed Ni. Inorganic Crystal Structure Database (ICSD) standards for BDD (21536), Ni (8688) and Cu (7954). b) High resolution, background subtracted, X-ray photoelectron spectra in the Ni 2p and Cu 3p regions.

To determine accurately the lattice parameters and crystallite sizes, the XRD pattern of the dealloyed electrode was fitted between 40–55° to three Lorentzian peaks associated with BDD (111), Ni (111) and (200) as displayed in Figure S5. The average lattice constant was found to be *a* = 0.351 nm with an average crystallite size of 22 nm, calculated with the Scherrer equation. The lattice constant of nickel is typically given as 0.352 nm,^51^ showing agreement with the results here.

XPS spectra associated with Cu and Ni 2p orbitals before and after dealloying are illustrated in Figure 4b. Qualitative analysis of the spectra show the substantial decrease of the Cu 2p signal with respect to Ni 2p after the dealloying step. The spontaneous formation of oxides and hydroxides in ambient conditions prevents any meaningful analysis of Cu and Ni speciation,^52,53^ however, we integrated the signals and calculated elemental ratios taking into account the corresponding sensitivity factors. Table 1 contrasts the Ni/(Cu+Ni) atomic ratio obtained from XPS and EDS. The results show that there is an overall Ni excess in the bulk of the as-deposited films, while the surface composition is close to 1:1 as estimated from XPS. Interestingly, EDS analysis of the Ni atomic ratio in cross sections of as-deposited films (see Figure S6) shows a compositional gradient where Ni increases from approximately 50% at the film surface to 90% at the junction with BDD. This is a very important observation, revealing a preferential deposition of Ni over Cu at the BDD surface. This Ni enrichment may also be responsible for the adhesion of the electrodeposited layer. Computational studies have concluded that Ni binding to O-terminated diamond surfaces is two times stronger than Cu (measured with a work of separation).^54,55^ After dealloying, the Ni ratio increases to above 90% with respect to Cu in the bulk and at the surface, leading to the formation of the highly corrugated electrodeposited films shown in Figure 3.

**Table 1:** Compositional Information of Electrodeposited NiCu before and after Dealloying^a^

	Ni composition / %	
	EDS	XPS
as-deposited NiCu	68.0±1.0	53±1
DA Ni	96.0±0.2	94±2

a Ni composition determined by (*x*_Ni_ / (*x*_Ni_ + *x*_Cu_)) × 100, where *x*_Ni_ and *x*_Cu_ refer to the relevant compositional value of Ni and Cu associated with each method. The EDS composition is the average of three locations and the error the standard deviation.

### Hydrogen Evolution Reaction Kinetics at Corrugated Ni Surfaces on BDD

The cyclic voltammogram in Figure 5a illustrates the characteristic pseudo-capacitive responses associated with the surface confined oxidation of Ni sites to α-Ni­(OH)_2_. As described in the experimental section, we use the charge associated with this to estimate the electrochemical surface area (*A*_ECSA_) and the roughness factor (RF = *A*_ECSA_ / *A*_geo_) of the films after the dealloying step. As shown in Table S1, the deposition at constant charge (1.0 C cm^–2^) has a significant effect on the RF of the dealloyed films. As discussed previously, the deposition of Cu is diffusion limited whereas Ni^2+^, present in much higher quantities, is under kinetic control. Therefore, decreasing deposition potential increases the rate of Ni deposition relative to Cu. We can also see that increasing the deposition charge at constant potential (−0.42 V) leads to a further increase in RF. It should be mentioned that the voltammogram in Figure 5a was obtained for a film with a RF of approximately of 30. Such highly corrugated films are characterized by a broad distribution of active sites, leading to broadening of the pseudo-capacitive responses in comparison to those obtained at more homogeneous surfaces.^56^ Figure S7 shows a voltammogram recorded at films with an RF of 11, this exhibits clearer defined pseudocapacitive features associated with α-Ni­(OH)_2_ formation. Figure 5b shows linear sweep voltammograms (LSV) obtained at DA Ni films deposited at various potentials, with a constant charge density (1.0 C cm^–2^) and at −0.42 V vs. RHE and various deposition charge densities. It can be observed the overpotential for a geometric current density (*j*_geo_) of 10 mA cm^–2^ (η_10_) has a stronger dependence on the deposition charge than the deposition potential. Depositions for a |*Q*_dep_| greater than 5.0 C cm^–2^ showed no improvements in η_10_ along with material loss, making the data irreproducible. Figure 5c illustrates the close correlation between η_10_ and *A*_ECSA_, suggesting that the observed changes in electrocatalytic performance are extrinsic in nature. However, as described further below, there are some subtle changes which reveal mechanistic insights.

**Figure 5:**
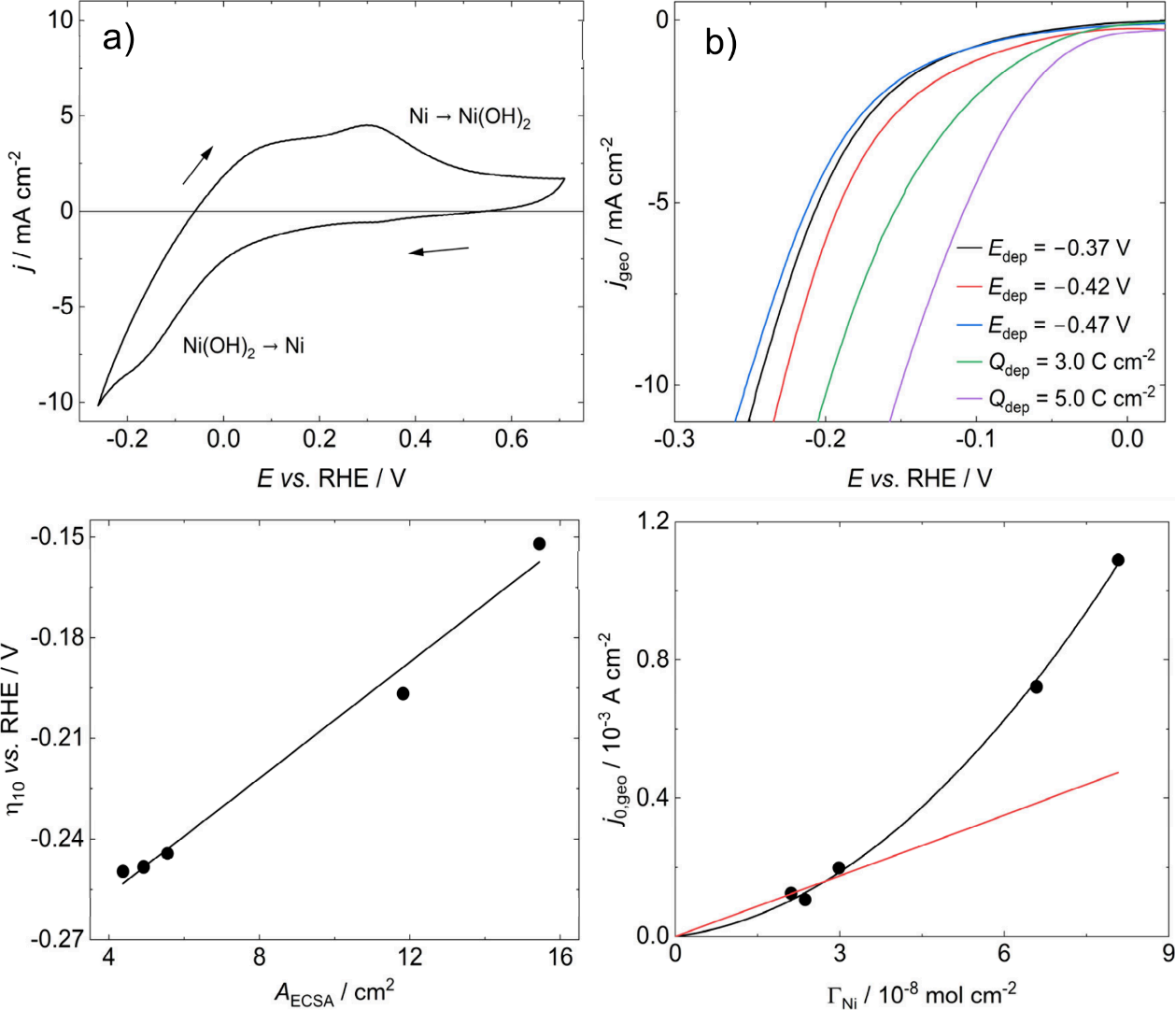
a) Typical voltammogram used to estimate the electrochemical surface area of dealloyed Ni electrodes, the charge associated with the oxidation of Ni to Ni­(OH)_2_ was used, ν = 50 mV s^–1^. b) Linear sweep voltammograms showing the effect of NiCu electrodeposition parameters on the HER activity following dealloying. pH13 KOH, ν = 5 mV s^–1^, CE: graphite rod, RE: Hg/HgO. c) Plot of the average η_10_ as a function of average *A*_ECSA_ for dealloyed Ni surfaces, showing the relationship between HER activity and surface area. d) Plot of exchange current density, *j*_0,geo_, as a function of the number of Ni active sites Γ_Ni_. The whole dataset was fit to a quadratic (black), and the points at *Q*_dep_ = 1.0 C cm^–2^ to a linear fit (red), both with intercepts set to 0, demonstrating that HER activity is 2nd order with respect to Ni. The linear fit was extrapolated across the whole Γ_Ni_ range for illustrative purposes.

Figure 5d shows the dependence of the geometric exchange current density, *j*_0,geo_, estimated from Tafel analysis of the LSV curves shown in Figure 5b, as a function of the Ni active sites, Γ_Ni_, estimated using Equation 2,

$$\Gamma_{\mathrm{Ni}}=\frac{Q_{\mathrm{Ni}\to \mathrm{Ni}{\left(\mathrm{OH}\right)}_{2}}}{nFA_{\mathrm{geo}}}$$ 2

where *n* was taken as 2. The underlying assumption here is that each electroactive Ni site is taking part in oxide phase formation. In this part of the analysis, the current-potential curves were extracted from LSV plots at 5 mV s^–1^, rather than steady state current measurements which are free from any capacitive or other dynamic interferences.^57^ However, the Tafel slopes obtained from our analysis are in the range 120 – 165 mV, which are consistent with reports in the literature.^58–61^ A more detailed analysis of Tafel slopes will be discussed at the end of the paper.

If the changes in the activity of corrugated Ni films are only extrinsic, then *j*_0,geo_ should have a linear dependence on Γ_Ni_. However, the trend in Figure 5d reveals that *j*_0,geo_ exhibits a square dependence on Γ_Ni_. This unexpected observation strongly suggests that the rate limiting step of the HER is 2^nd^ order with respect to the number of active Ni sites on the dealloyed Ni electrodes. To the best of our knowledge, this the first report that directly measures the effect the Ni surface area has on the rate of the HER in this way.

A plot of *j*_0,geo_ vs. Γ_Ni_^2^ was fitted to a linear regression (Figure S8), resulting in a gradient of 1.7×10^11^ A mol^–2^ cm^2^. Dividing through by *N*_A_ *F* then gives a value for the slope of 3×10^–22^ m^2^ s^–1^, which has the units of a diffusion coefficient. From this we can conclude that the rate law for HER at highly corrugated, dealloyed Ni deposits is showing that the process is controlled by the kinetics of surface diffusion and collision of adsorbed intermediates during the Volmer step. This value likely represents an average of the various intermediates, and furthermore the errors associated with the estimation of *j*_0_ and *Q*_Ni → Ni(OH)_2__ mean that this should be taken as approximate. The diffusion of H and O has been computationally studied at Ni single crystal surfaces in vacuum,^62,63^ and can be used to estimate values at 298 K on the order of 10^‑10^ m^2^ s^–1^ and 10^–27^ m^2^ s^–1^ for H and O respectively, where the diffusion of H and O on Ni differs widely because of the significantly lower activation energy for H diffusion. Interestingly, our estimate for intermediate diffusion during the Volmer step lies within these values.

To rationalize the second-order dependence of HER kinetics on the number density of Ni active sites, we need to consider that HER in alkaline electrolytes begins with the dissociation of water to form adsorbed hydrogen, H_ad_. This process known as the Volmer step is shown in Equation 3. Adsorbed hydrogen then recombines either electrochemically *via* the Heyrovsky step, or chemically with the Tafel, corresponding to Equation 4 and Equation 5 respectively.

H2O+e−⇌Had+OH−⁣Volmer(3)Had+H2O+e−⇌H2+OH−⁣Heyrovsky(4)2Had⇌H2⁣Tafel(5) 3

The conventional representation of the initial Volmer step (eq 3) indicates a first-order dependence of the reaction on the number of active sites. Recently, studies have demonstrated the importance of OH adsorption during water dissociation and its role in determining the rate of HER.^64–66^ The slow desorption of OH can have a blocking effect, limiting the availability of active sites to perform HER. Furthermore, the presence of simultaneously adsorbed H_2_O, H and OH has been recently observed with *in situ* Raman spectroscopy under HER conditions at Ru surfaces in 0.1 M NaOH.^67^ Whilst it rarely appears to be discussed, the corollary of this is that a more accurate description of water dissociation should be eq 6, where M* is an active site on the electrode, with the subsequent desorption of adsorbed OH occurring *via* eq 7. Now, as can be seen, a second-order dependence on the metal site can be expected.

$$H_{2}O+2M^{*}⇌M-H_{\mathrm{ad}}+M-OH_{\mathrm{ad}}$$ 6

$$M-OH_{\mathrm{ad}}+e^{-}⇌{\mathrm{OH}}^{-}$$ 7

Ni as a good binder of OH and promoter of water dissociation is recognized,^2,64^ and this is supported by numerous computational studies,^68–70^ which supports our experimental evidence that eq 6 is operative on dealloyed Ni surfaces.

Figure 6 shows the Tafel plot of a DA Ni catalyst on BDD, along with a polished Ni disc measured for comparison. The presence of two Tafel slopes can be observed at low and high overpotentials, labelled *b*_1_ and *b*_2_, respectively. Figure S9 shows the same results normalised by the ECSA. The linear fits for each region are given in Table 2, where *j*_0,ECSA_ is the exchange current densities normalised by the electrochemical surface area. For the Ni disc, parameters of *b* = 127 mV, *j*_0,geo_ = 6.6 μA cm^–2^, *j*_0,ECSA_ = 5.5 μA cm^–2^ were calculated. At high pHs, a single Tafel slope of ∼120 mV and *j*_0_ of 1–10 μA cm^–2^ has been reported several times in the literature for Ni electrodes,^58,59,71–73^ which is consistent with a Volmer–Heyrovsky mechanism with a Volmer rate determining step, as previously shown in Equations 3–5.

**Figure 6:**
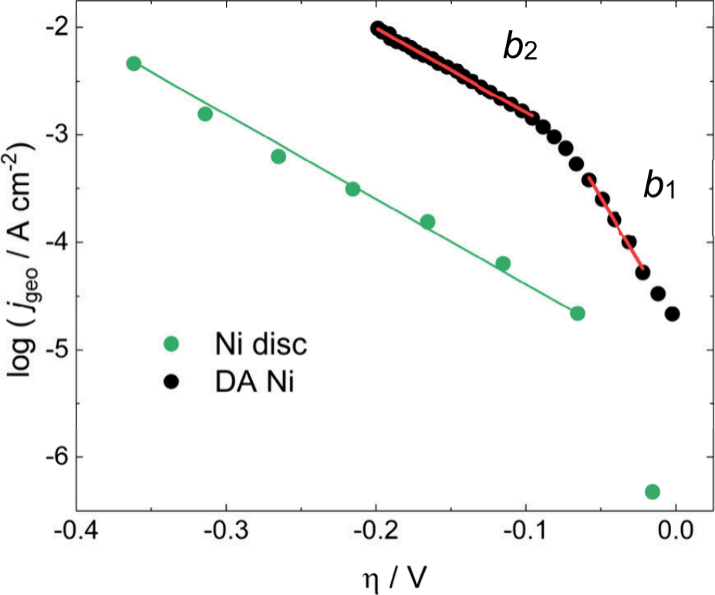
Tafel plots of a DA Ni catalyst deposited at −0.42 V vs. RHE for 5.0 C cm^–2^, and *r* = 0.25 mm Ni disc in pH 13 KOH at 298 K.

**Table 2:** Tafel Parameters of Electrochemically DA Ni Electrodes on a BDD Substrate and a Ni Disc Obtained in pH 13 KOH at 298 K

	Slope 1			Slope 2		
	*j*_0,geo_ / μA cm^–2^	*j*_0,ECSA_ / μA cm^–2^	*b* / mV	*j*_0,geo_ / μA cm^–2^	*j*_0,ECSA_ / μA cm^–2^	*b* / mV
DA Ni	17±1	0.8±0.1	40±3	352±28	17±2	137±7
Ni disc	-	-	-	6.6±0.7	5.5±0.6	127±2

On the other hand, an initial slope of ∼40 mV followed by a transition to a slope approaching 118 mV is characteristic of a Volmer-Heyrovsky mechanism where the Heyrovsky step is now rate limiting.^74^ This occurs when the rate of the Volmer step is significantly faster than the Heyrovsky. At low overpotentials the coverage of H_ads_ is also low. As the overpotential increases, so does the coverage of H_ads_ since it is being supplied faster than it can recombine, until it becomes high enough that the Volmer step transitions to being rate limiting and this is where the inflection point is observed in the Tafel plots. Figure 6 indicates that this is taking place at the dealloyed Ni electrodes, suggesting a change in the HER mechanism and, when considering the calculated Tafel parameters of the Ni disc electrode, an additional increase in the intrinsic activity of the catalyst relative to metallic Ni. It should be mentioned that the presence of two Tafel slopes in HER at Ni alloys have also been observed, although the rationale remains to be established.^75,76^ Conway *et al.* electrodeposited Ni:Mo:Cd (79:20:1 at. %) electrodes,^77,78^ and reported Tafel slopes of 30-38 mV at low overpotentials, followed by 120-125 mV at higher overpotentials, when η > 0.1 V, in 1 M NaOH at 298 K, which was attributed to the formation of a hydride phase during the electrodeposition process. However, other works have concluded that nickel hydride deactivates the electrode, increasing in the Tafel slope.^79,80^

As shown in Table 2, *j*_0,geo_ for the DA Ni film is approximately 60 times higher than at Ni discs, which decreases to a factor of 3 when considering the roughness factor (*j*_0,ECSA_). This observation suggests a higher intrinsic HER activity in highly corrugated DA Ni films. Several articles have also reported enhancement of intrinsic activity at DA Ni electrocatalysts, although the rationale remains to be fully elucidated.^81–83^ As discussed above, *j*_0,geo_ scales to the square of the number density of Ni sites (see Figure 5d), which could explain why highly corrugated films exhibits a higher *j*_0,ECSA_ (given that ECSA scales linearly with Γ_Ni_). However, we cannot fully rule out strain and electronic effects that may arise from traces of Cu and the high degree of corrugation, as reported on highly active Pt core-shell nanoparticles for example.^84–86^ Savinova and co-workers have synthesised NiCu alloys, with 95:5 at. % (similar to the surface composition of the DA Ni electrodes) and studied their HER/HOR activity.^81,87,88^ The addition of Cu enhanced the activity of both reactions over metallic Ni and, with the assistance of microkinetic modelling, this was attributed to an acceleration of the Volmer step and a lowering of the H adsorption energy. H adsorbs weakly onto Cu, which could attenuate its stronger adsorption to Ni sites.^12^ Santos *et al.* additionally performed computational calculations on a similar system composed of Ni(111) covered by a Cu monolayer,^89^ predicting improvements in HER activity by lowering the activation barrier for the Volmer step, without changing the d band structure. Further studies will be required to confirm these hypotheses.

## Conclusions

This report describes, for the first time, the electrodeposition of continuous highly corrugated Ni films onto BDD for their exploitation as cathodes for alkaline water electrolysis. NiCu alloys electrodeposited onto O-terminated BDD exhibited a composition gradient, with Ni-rich content at the BDD junction and a Cu-rich top layer. This configuration enables the formation of highly corrugated films upon electrochemical dealloying, with a Ni atomic ratio of 95% across the electrocatalytic layer. For the first time, we show that the phenomenological HER exchange current density (normalised by geometric area) exhibits a second-order dependence on the number of Ni active sites evaluated from the charge of the α-Ni­(OH)_2_ redox transition. This observation was rationalized based on surface water activation leading to OH formation (Volmer step) in alkaline solutions. Furthermore, Tafel analysis of the most active dealloyed electrode indicates a modification of the hydrogen evolution mechanism, from the conventional Volmer–Heyrovsky mechanism limited by water dissociation, to one where the Heyrovsky step is now rate limiting at low overpotentials. Finally, our study opens a new avenue for designing dimensionally stable electrodes for electrochemical transformations. Indeed, recent developments on CVD growth of BDD films enable their implementation as thin functional coatings on a variety of industrially relevant materials.^90^

## Supplementary Material



## Data Availability

Data are available at the University of Bristol data repository, data.bris, at https://doi.org/10.5523/bris.1g1vkjpyz8oj12kd9tgl01o3a1.

## Notes

Figure S1 SEM images of BDD electrodes after electrolysis; Figure S2 typical transients of NiCu electrodeposition and Cu dealloying, Figure S3 low magnitude cross-sectional SEM images of electrodeposited NiCu and dealloyed Ni; Figure S4 SEM images of dealloyed Ni showing the effect of initial deposition charge density; Figure S5 XRD pattern of dealloyed Ni and peak fits; Figure S6 cross-sectional SEM image of NiCu and EDS linescan; Figure S7 voltammogram of α-Ni­(OH)_2_ formation and reduction on a DA Ni electrode RF = 11; Table S1 roughness factors and HER activity of dealloyed Ni; Figure S8 plot of *j*_0,geo_ as a function of Γ_Ni_^2^; Figure S9 Tafel plots of an Ni disc and dealloyed Ni (PDF)
