# Supplementary material for: Highly Corrugated Ni Films Electrodeposited onto Boron Doped Diamond Electrodes for Alkaline Water Electrolysis
Source: ACS Electrochem. 2025 Oct 20;1(11):2591–601. doi: 10.1021/acselectrochem.5c00319 (PMC12598865; doi:10.1021/acselectrochem.5c00319)
Supplement: Supplementary file 1 [file ec5c00319_si_001.pdf]

Supplementary Information

Highly Corrugated Ni Films Electrodeposited onto Boron Doped Diamond Electrodes for Alkaline Water Electrolysis

Alexander W. Black, Paul W. May, David J. Fermin*

School of Chemistry, University of Bristol, Bristol, BS8 1TS, UK

*Corresponding author. Email: David.Fermin@bristol.ac.uk

Table of Contents

Figure S1: SEM images of BDD electrodes after electrolysis

Figure S2: typical transients of NiCu electrodeposition and Cu dealloying

Figure S3: low magnitude cross-sectional SEM images of electrodeposited NiCu and dealloyed Ni

Figure S4: SEM images of dealloyed Ni showing the effect of initial deposition charge density

Figure S5: XRD pattern of dealloyed Ni and peak fits

Figure S6: cross-sectional SEM image of NiCu and EDS linescan

Figure S7: voltammogram of α-$Ni(OH)_2$ formation and reduction on a DA Ni electrode RF = 11

Table S1: roughness factors and HER activity of dealloyed Ni

Figure S8: plot of $j_{0,geo}$ as a function of Γ_{Ni}^2

Figure S9: Tafel plots of an Ni disc and dealloyed Ni

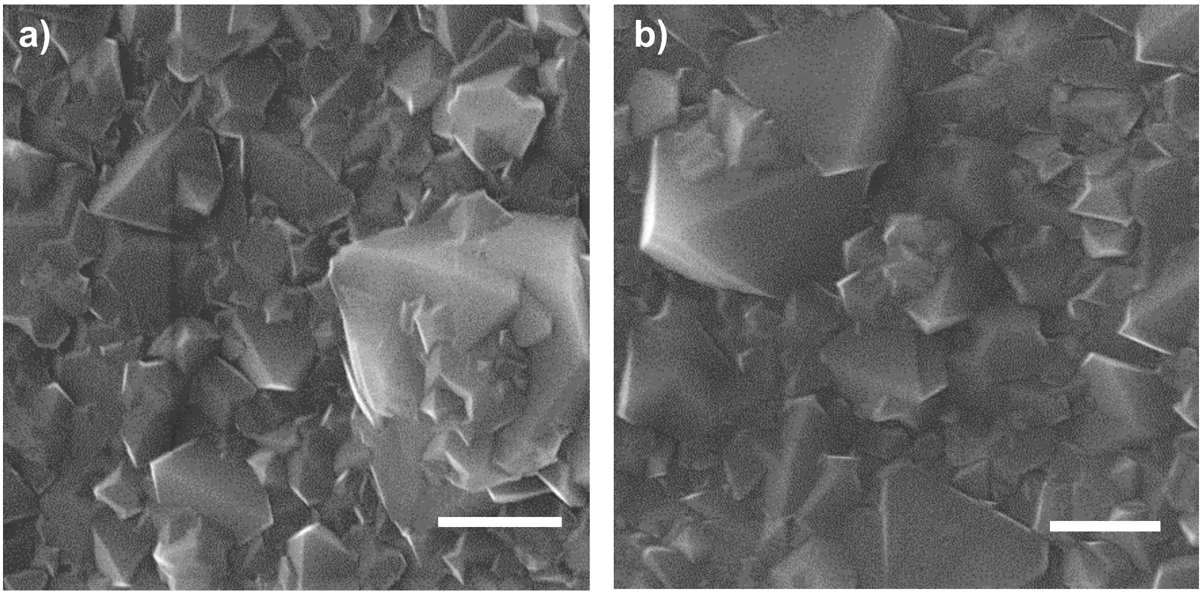

Figure S1: representative SEM images of BDD electrodes after electrolysis at a) -10 mA cm^{-2}, b) +10 mA cm^{-2} in pH 13 KOH at 25°C for 8 hours. Scale bar represents 2 µm.

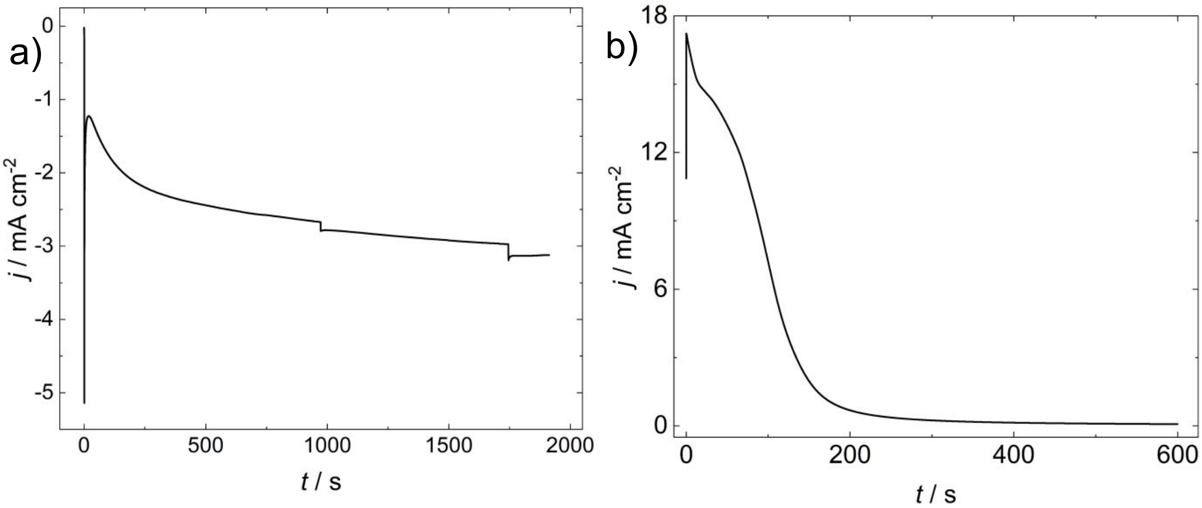

Figure S2: typical transients of a) NiCu electrodeposition at -0.42 V *vs*. RHE for 5 C cm^{-2} Cu dealloying at 1.03 V vs. RHE. WE: *r* = 4 mm BDD, CE: glassy carbon, RE: Ag/AgCl.

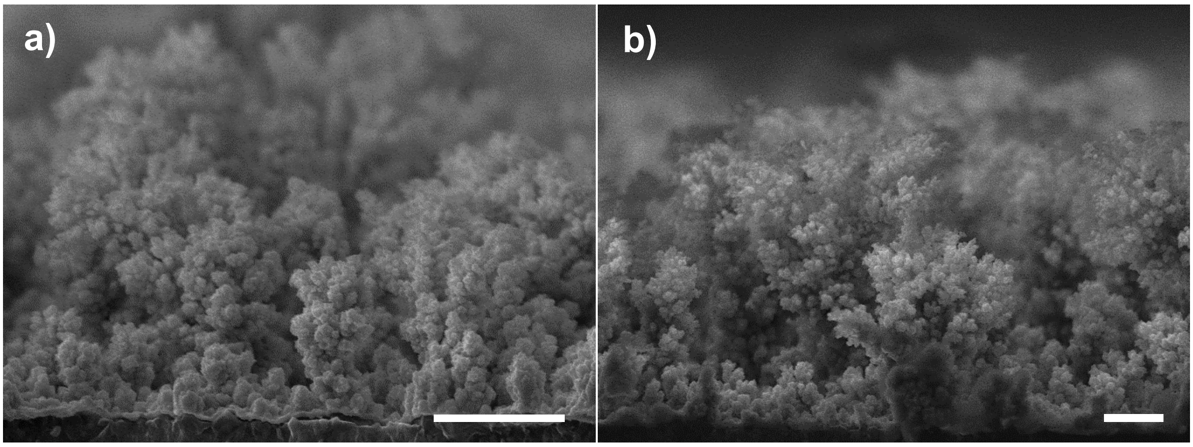

Figure S3: low magnification cross-sectional SEM images of NiCu alloys electrodeposited onto BDD a) as deposited, b) after dealloying. Scale bar represents 5 μm.

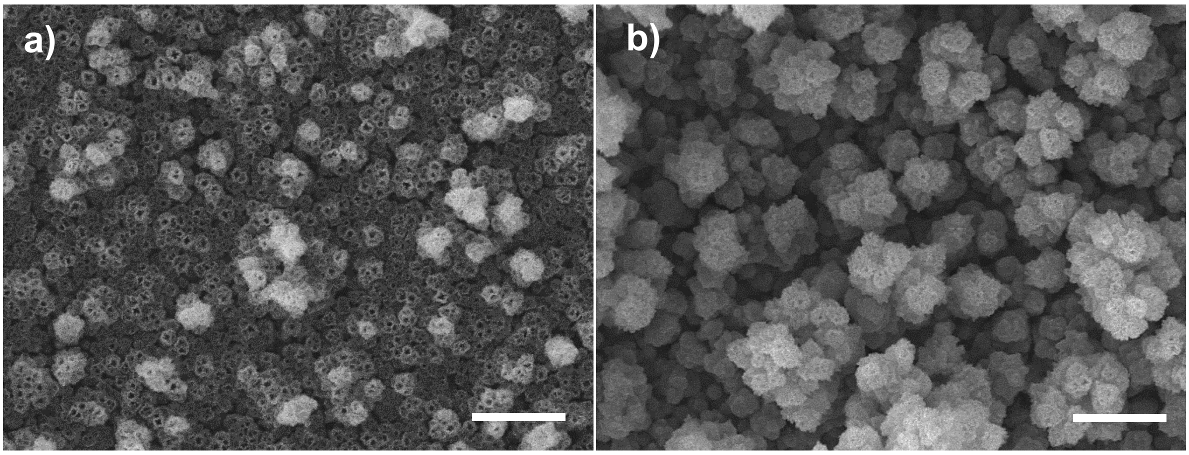

Figure S4: SEM images of dealloyed Ni on BDD showing the effect of initial deposition charge, with a) $|Q_{dep}|$ = 1 C cm^{-2}, b) $|Q_{dep}|$ = 3 C cm^{-2}. Scale bar represents 2 μm.

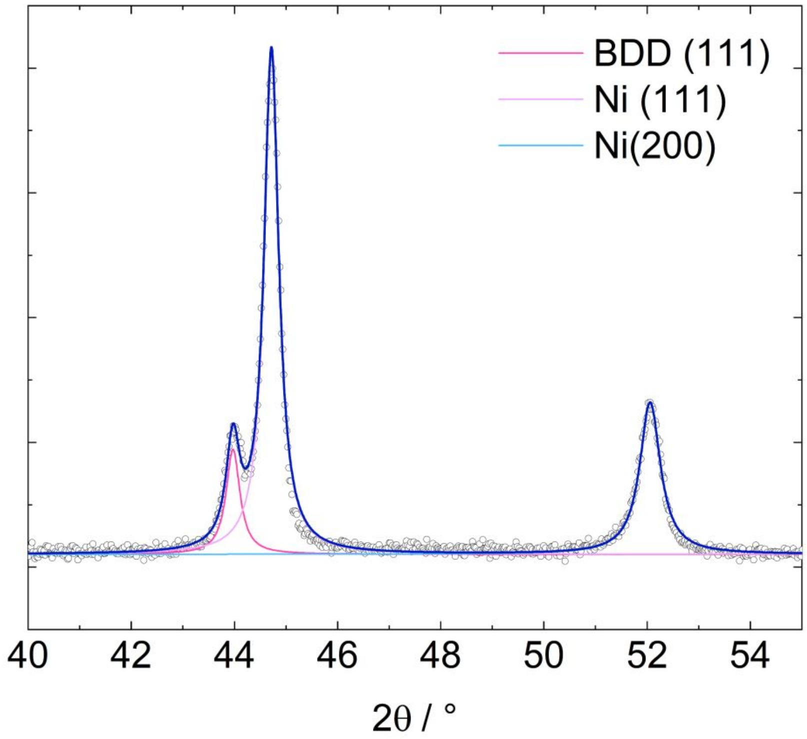

Figure S5: XRD pattern of dealloyed Ni deposited onto BDD, fitted to three Lorentzian environments corresponding to the BDD (111), Ni (111) and Ni (200) reflections.

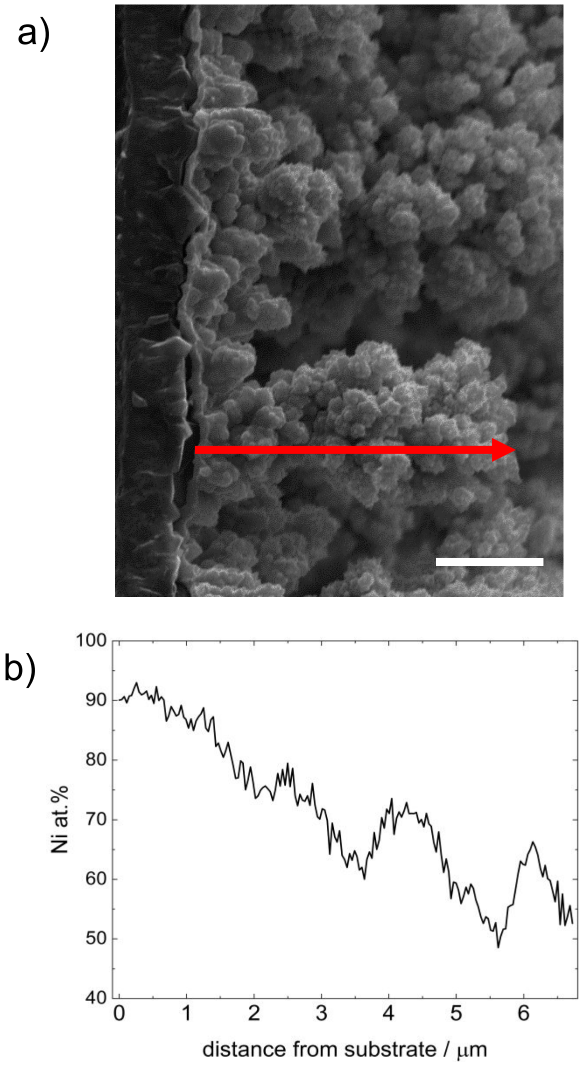

Figure S6: a) representative SEM image and, b) accompanying EDS linescan of as-deposited NiCu onto BDD showing change in composition with distance. Red arrow indicates direction of scan. Scale bar represents 2 μm.

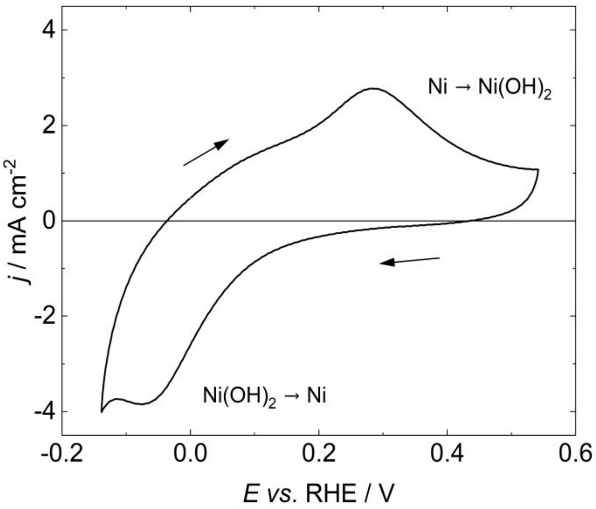

Figure S7: typical voltammogram showing the formation of α-$Ni(OH)_2$ and its reduction to Ni in pH 13 KOH. E_{dep} = -0.42 V *vs.* RHE, Q_{dep} = 1.0 C cm^{-2}, v = 50 mV s^{-1}.

Table S1: The effect of deposition potential and deposition charge density from a bath containing 0.2 M $NiSO_4$ and 0.01M $CuSO_4$ onto a *r* = 4 mm BDD WE on the roughness and HER activity of the deposited material. RF measured using the α-$Ni(OH)_2$ method. RF and η_{10} values are the average of three repeats and the error the standard deviation.

E_{dep} *vs*. RHE / V	$\lvert Q_{dep}\rvert$ / C cm^{-2}	RF	η_{10} / V
-0.37	1.0	9.8±0.7	-0.248±0.001
-0.42	1.0	11.4±0.1	-0.237±0.008
-0.47	1.0	8.7±0.6	-0.250±0.003
-0.42	3.0	23.5±0.8	-0.197±0.006
-0.42	5.0	30.7±0.3	-0.152±0.004

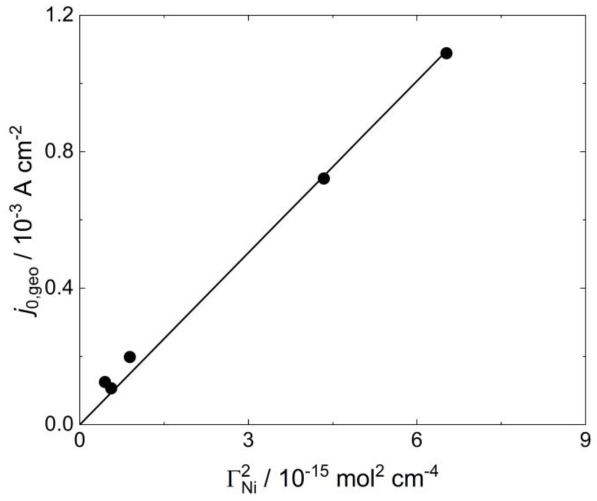

Figure S8: plot of the exchange current density, $j_{0,geo}$ as a function of the square of the number of Ni active sites, Γ_{Ni}^2 for DA Ni catalysts.

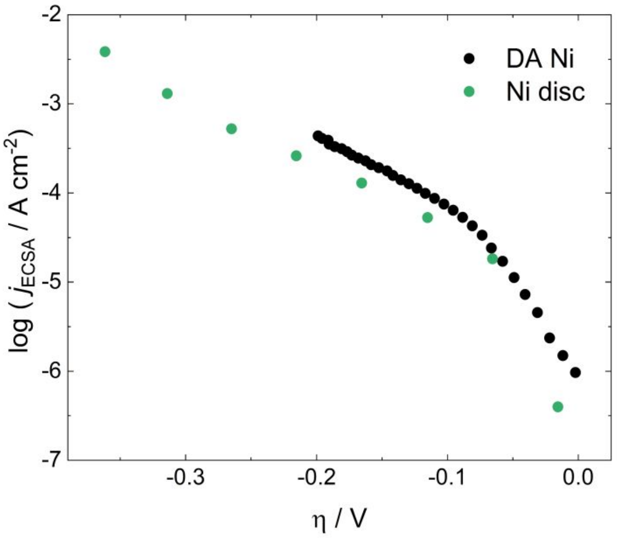

Figure S9: Tafel plots of an *r* = 0.25 mm Ni disc and *r* = 4 mm dealloyed Ni electrode in pH 13 KOH at 298 K where currents are normalised by electrochemical surface area
